# The Effects of Time on Task in Response Selection - An ERP Study of Mental Fatigue

**DOI:** 10.1038/srep10113

**Published:** 2015-06-09

**Authors:** Tina Möckel, Christian Beste, Edmund Wascher

**Affiliations:** 1 Leibniz Research Centre for Working Environment and Human Factors, Ardeystr. 67, 44139 Dortmund, Germany; 2 Cognitive Neurophysiology, Department of Child and Adolescent Psychiatry, Faculty of Medicine of the TU Dresden, Fetscherstr. 74, 01307 Dresden, Germany

## Abstract

Long lasting involvement in a cognitive task leads to mental fatigue. Substantial efforts have been undertaken to understand this phenomenon. However, it has been demonstrated that some changes with time on task are not only related to mental fatigue. The present study intends to clarify these effects of time on task unrelated to mental fatigue on response selection processes at the behavioural and electrophysiological level (using event-related potentials, ERPs). Participants had to perform a Simon task for more than 3 hours and rated their experienced mental fatigue and motivation to continue with the task at several time points during the experiment. The results show that at the beginning of the experiment some unspecific modulations of training and adaptation are evident. With time on task participants’ ability to resolve response conflict appears to become impaired. The results reveal that time on task effects cannot be completely explained by mental fatigue. Instead, it seems that an interplay of adaptation at the beginning and motivational effects in the course of a task modulate performance and neurophysiological parameters. In future studies it will be important to account for the relative contribution of adaptation and motivation parameters when time on task effects are investigated.

Long lasting involvement in a cognitive task leads to deficits in attention, working memory and action control. This phenomenon is termed mental fatigue. Since mental fatigue may substantially increase the risk of accidents it is an important human factor of influence in various fields of applied research^1^,^2^. Therefore, substantial efforts have been undertaken to characterize this phenomenon behaviourally^3^,^4^ and in terms of neurophysiological measures^5^,^6^. However, recent studies on detailed time course analyses^7^ and on task adaptation^8^ have demonstrated that some changes with time on task are not only related to mental fatigue. The present study intends to clarify the effects of time on task in a well-established setting of a long-lasting cognitive task.

Experimental studies demonstrate that subjects who suffer from mental fatigue may have problems in keeping their attention focused on their current task or on the relevant information^9^,^10^,^11^ reported impaired executive control processes like planning and mental flexibility with mental fatigue. In terms of further cognitive experiments mental fatigue leads to insufficient information processing and in consequence to increased error rates, slowing of responses and deterioration of behavioural adjustments^9^,^12^,^13^,^14^.

On a neurophysiological level these changes in information processing are assumed to be reflected in event-related potentials (ERPs) of the electroencephalogram (EEG). Different studies showed an association of action control and response conflict evaluation with the N2, which is a negative wave mainly centred at fronto-central locations^15^,^16^,^17^. In a long-lasting choice response task 6 demonstrated that the ability to process incongruencies becomes impaired by mental fatigue. This deterioration of performance was accompanied by a change in N2 amplitude, meaning that at the beginning of the experiment the N2 amplitude was larger in conflict trials than in trials where there was no response conflict. Interestingly, this discrepancy (i.e. conflict effect) decreased with time on task, leading to the suggestion that the N2 amplitude is reflecting the declining ability to perform response selection in conflicting situations. The P3, a late positive wave on parieto-occipital locations, is another ERP component that is often linked to the identification and evaluation of stimuli^18^,^19^^.^^6^ showed that increases in response times and error rates with time on task were accompanied by increases in P3 latency. They proposed that this might reflect a change in the strategy of information processing from a complex but effortful evaluation of received information to a more passive and long-lasting one. This way participants may need more time but less effort for the evaluation of incoming signals. Additionally,^20^ found a linear decrease of P3 amplitude with time on task, which they interpreted as a result of a decrease in vigilance.

However, interpretations of changes in performance and in neurophysiological parameters with time on task as correlates of mental fatigue have to be treated with caution. Especially in early phases of an experiment training and adaptation mechanisms may lead to a modulation of electrophysiological parameters that are comparable to changes, which are associated with mental fatigue^8^. Unexpected time courses were also shown for more common indicators of mental fatigue in the frequency domain of the EEG.^7^ revealed that an increase in alpha power cannot be assigned to increasing mental fatigue, as was commonly assumed^21^,^22^, because it only varies at the beginning of a long-lasting experiment. It appears that both early changes, due to adaptation or training, as well as late changes, due to mental fatigue, may contribute to the question of what happens when people are exposed to longer-lasting cognitive tasks. However, those factors might be easily missed whenever the duration of an experiment is restricted or when there is no continuous extraction of behavioural and physiological measures.

In the present study we tried to investigate the effects of mental fatigue on behaviour and ERPs by letting participants perform a spatial stimulus-response correspondence (Simon) task for more than 3 hours. In this task the irrelevant spatial location of a target stimulus might either accelerate responses when it is corresponding to the response location or decelerate responses when non-corresponding. Thus, the Simon task requires substantial response control due to variation in spatial stimulus-response correspondence^23^,^24^,^25^,^26^. The effects of mental fatigue can be nicely observed with this task^6^. In contrast to the latter study we used a highly structured experimental design by dividing the experiment in 3 equal blocks and each block in 3 equal sub-blocks so that a detailed analysis of the time course was possible. We additionally added short breaks after every block to examine the specific effects of short interruptions on performance. At several time points during the experiment participants provided subjective ratings to control for subjective feelings of mental fatigue and motivation. This way we intended to dissociate between adaptation effects and true effects of mental fatigue.

## Results

### Ratings

**Figure 1:**
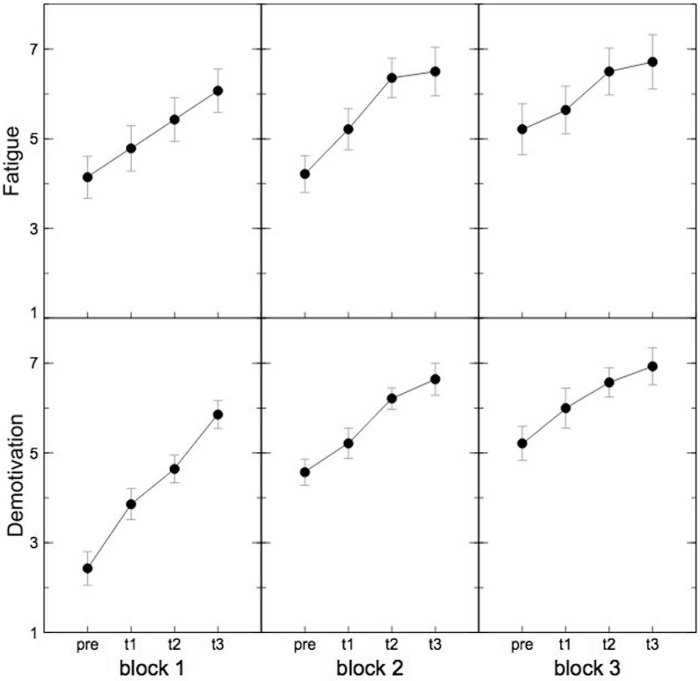
Subjective ratings. The upper graph shows the subjective rated mental fatigue during the experiment on a 9-step Likert scale. The bottom graph shows the subjective rated motivation to continue with the task during the experiment on a 9-step Likert scale. High mental fatigue or low motivation to continue with the task respectively are represented by high numbers. There is a significant increase of mental fatigue and and a significant decrease of motivation with time on task within the blocks. As error bars standard errors were used.

Within blocks subjectively perceived mental fatigue increased reliably, as a significant main effect was found for the factor SUBBLOCK (F(3,39) = 26.09, p < .001, *η*^2^_p_ = .67). Post hoc tests showed a steady increase of mental fatigue ratings over the first 3 time points within a block (for all p < .05). Further, post hoc comparisons between the last rating of a block and the first rating of the following block showed a recovery after the breaks (F(1,13) = 45.77, p < .001, *η*^2^_p_ = .78 and F(1,13) = 20.25, p <. 01, *η*^2^_p_ = .61 respectively). Comparing the first rating of the first block with the first ratings of the other blocks revealed that the recovery was almost complete (for all p > .1; see Fig. 1).

The motivation ratings showed a slightly different pattern. The ANOVA revealed a main effect for the factor BLOCK (F(2,26) = 29.90, p < .001, *η*^2^_p_ = .70). Post hoc comparisons showed that the first block differed significantly from the others (for both p < .001) and the second block marginally from the third block (p < .1). This indicates a continuous decrease of motivation with time on task. Furthermore, there was a significant main effect for the factor SUBBLOCK (F(3,39) = 51.61, p < .001, *η*^2^_p_ = .80). In contrast to the ratings of mental fatigue, motivation steadily decreased across all time points (for all p < .01). The ANOVA further showed a significant interaction BLOCK X SUBBLOCK (F(6,78) = 3.17, p = .030, *η*^2^_p_ = .20). Post hoc tests revealed that there was a significant decrease of motivation over the sub-blocks within all blocks (F(3,39) = 39.60, p < .001, *η*^2^_p_ = .75, F(3,39) = 16.36, p < .001, *η*^2^_p_ = .56 and F(3,39) = 11.75, p < .001, *η*^2^_p_ = .47 respectively). Figure 1 shows that the interaction may be explained by a stronger decrease of motivation during the first compared to the second and third bock. This is also underlined by the effect sizes (*η*^2^_p_) for the different ANOVAs (see above). Again, strong recovery after the breaks was visible (F(1,13) = 43.88, p < .001, *η*^2^_p_ = .77 and F(1,13) = 24.07, p < .001, *η*^2^_p_ = .65 respectively). However, the ratings did not completely reach the level of the beginning (F(1,13) = 32.50 _p_ < .001, *η*^2^_p_ = .714 and F(1,13) = 38.847, p < .001, *η*^2^_p_ = .75 respectively; see Fig. 1).

### Behaviour

**Figure 2:**
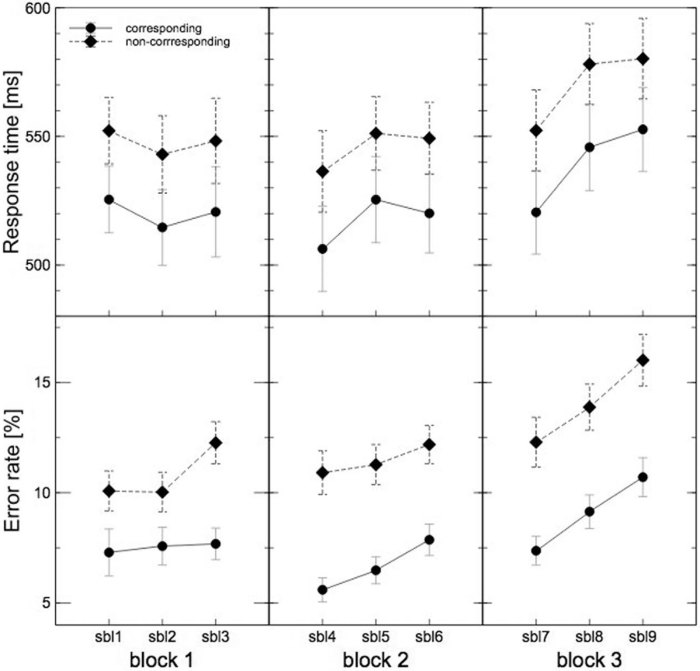
Behavioural performance. The upper graph shows the response times and the bottom graph the error rates in the course of the experiment. There is a significant increase of response times with time on task. Additionally, there is a significant Simon effect for both behavioural measurements, thus that participants show faster response times and less errors for corresponding compared to non-corresponding trials. For the error rates this effect becomes more pronounced with time on task. As error bars standard errors were used.

Response times for corresponding trials were significantly shorter compared to non-corresponding trials (CORRESPONDENCE: F(1,13) = 38.18, p < .001, *η*^2^_p_ = .75). Thus, a regular Simon effect was obtained that did not vary with any parameter of time on task. Furthermore, the ANOVA for the response times revealed a significant interaction of the factors BLOCK X SUBBLOCK (F(4,52) = 7.28, p < .001, *η*^2^_p_ = .36). Post hoc tests showed a marginal significant increase with time on task (BLOCK: F(2,26) = 5.18, p = .070, *η*^2^_p_ = .22). Additionally, the response times over the sub-blocks within the first and third block revealed a marginal variation (F(2,26) = 5.94, p < .1, *η*^2^_p_ = .31, F(2,26) = 3.51, p = n.s. and F(2,26) = 6.01, p < .1, *η*^2^_p_ = .32 respectively), indicating (as can be seen in Fig. 2) that within the first block the response times decreased, whereas there was an increase in response times within the third block.

Participants committed less errors within the corresponding trials compared to the non-corresponding trials (CORRESPONDENCE: F(1,13) = 18.62, p < .001, *η*^2^_p_ = .59). Thus, also for the error rates the regular pattern for this task was obtained. Further, a significant interaction of the factors BLOCK X CORRESPONDENCE was shown (F(2,26) = 5.08, p = .021, *η*^2^_p_ = .28). Post hoc comparisons revealed that the difference in error rates between corresponding and non-corresponding trials increased with time on task (F(1,13) = 7.69, p < .05, *η*^2^_p_ = .37, F(1,13) = 25.13, _p_ < .001 *η*^2^_p_ = .66 and F(1,13) = 20.05, p < .001, *η*^2^_p_ = .61 respectively; see Fig. 2).

### ERP

**Figure 3:**
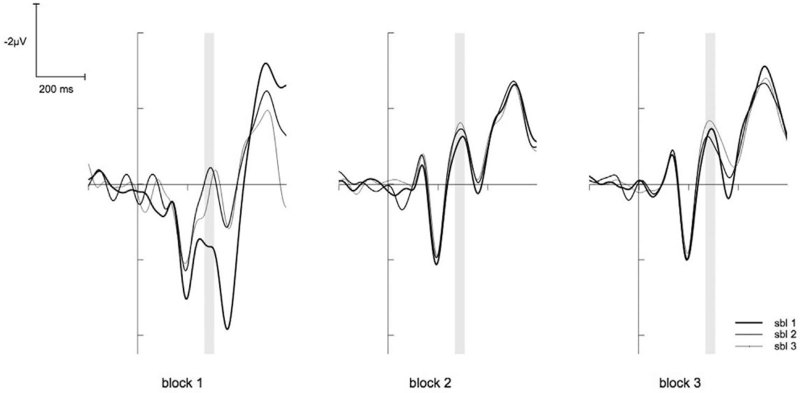
The N2 on FCz. The graph shows the N2 on FCz for the trials for the 9 sub-blocks (sbl). The time window of the N2 is marked by the grey underlining. The N2 amplitude increases with time on task but this effect is mostly explained by a severe increase from sub-block 1 to sub-block 2.

**Figure 4:**
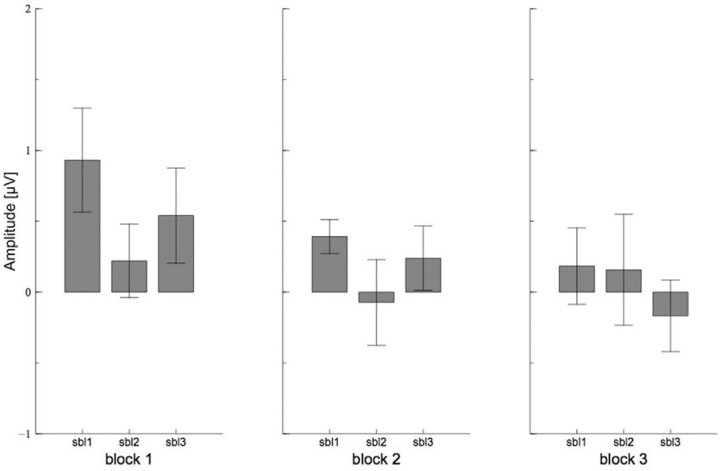
The N2 difference for non-conflict versus conflict trials on FCz. The graph shows the decreasing difference in N2 amplitude between corresponding and non-corresponding trials with time on task on FCz. As error bars standard errors were used.

The ANOVA for the N2 showed a significant main effect for the factor BLOCK (F(2,26) = 14.58, p < .001, *η*^2^_p_ = .53). Post hoc tests revealed that the first block differed significantly from the other blocks (for both p < .01), indicating less pronounced N2 amplitude at the beginning of the task. Another significant main effect was found for the factor SUBBLOCK (F(2,26) = 14.17, p < .001, *η*^2^_p_ = .52). Post hoc tests revealed that the first sub-block differed significantly from the other sub-blocks (for both p < .05), revealing that the N2 amplitudes were smaller during the first compared to the second and third sub-block. However, post hoc tests for the significant interaction of BLOCK X SUBBLOCK (F(4,52) = 11.09, p < .001, *η*^2^_p_ = .46) showed that the N2 amplitude for the sub-blocks varied only within the first block (F(2,26) = 20.47, p < .001, *η*^2^_p_ = .61, F(2,26) = .72, p = n.s. and F(2,26) = .94, p = n.s. respectively). This suggests that the most prominent increase of the N2 amplitude from the first to the next sub-block took place during the first block (see Fig. 3). Additionally, the ANOVA showed a significant interaction of the factors BLOCK X CORRESPONDENCE (F(2,30) = 3.92, p = .040, *η*^2^_p_ = .23). Post hoc tests revealed that in the first block amplitudes were marginally more pronounced for the non-corresponding compared to the corresponding trials, but this difference vanished for the second and third block (F(1,13) = 6.00, p < .1, *η*^2^_p_ = .32, F(1,13) = 2.17, p = n.s. and F(1,13) = .08, p = n.s. respectively; see Fig. 4).

**Figure 5:**
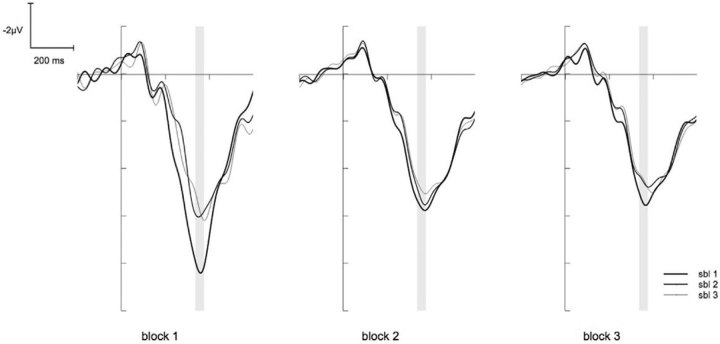
The P3 on Pz. The graph shows the P3 on Pz for the trials for the 9 sub-blocks (sbl). The time window of the P3 is marked by the grey underlining. The P3 amplitude decreases with time on task, but this effect is mostly explained by a severe decrease from sub-block 1 to sub-block 2.

The ANOVA for the P3 showed a similar result pattern. There was a significant main effect for the factor BLOCK (F(2,26) = 6.78, p = .018, *η*^2^_p_ = .34). The post hoc tests showed that the first block differed significantly from the second block and marginally from the third block (p < .05 and p < .1 respectively), indicating a decrease of the P3 amplitude with time on task. Another significant main effect was found for the factor SUBBLOCK (F(2,26) = 33.76, p < .001, *η*^2^_p_ = .72). The post hoc tests showed that the first sub-block differed significantly from the second and the third sub-block (for both p < .001), indicating a decrease of P3 amplitude at the beginning of each block. Additionally, the ANOVA revealed a significant interaction of the factors BLOCK X SUBBLOCK (F(4,52) = 3.21, p = .041, *η*^2^_p_ = .20). Post hoc tests showed that the amplitudes differed significantly across the sub-blocks within the first and third block (F(2,26) = 19.79, p < .001, *η*^2^_p_ = .60, F(2,26) = 4.11, p = n.s. and F(2,26) = 13.55, p < .001 *η*^2^_p_ = .51 respectively; see Fig. 5).

### Correlations

The mean of the motivation ratings correlated significantly with the error rates in the third block (r = .61, p = .020), indicating that at the end of the experiment a low motivation to continue with the task was accompanied by high error rates. Further correlations revealed that this effect increased over the three sub-blocks within this block (r = .48, p = .083, r = .49, p = .073 and r = .52, p = .055 respectively).

In addition, in the second block high error rates were accompanied by high P3 amplitudes (r = .54, p = .044). Further correlations showed that error rates tended to correlate with the P3 amplitude only in the second sub-block of the second block (r = .46, p = .096) and therefore revealed no clear pattern.

In the last block low N2 amplitudes were accompanied by high response times (r = .62, p = .018). Further analyses revealed that this effect increased within the block with time on task (r = .52, p = .054, r = .64, p = .014 and r = .68, p = .007 respectively).

## Discussion

The involvement in a cognitive task for a longer period of time may lead to learning and adaptation effects, to changes in the motivation to continue with the task or to so-called mental fatigue, a cognitive state that goes along with slowed information processing and increased error proneness^5^. Interestingly, even short interruptions of a long-lasting cognitive task may lead to substantially changes in those effects^27^. Thus, investigating the changes of time on task upon human behaviour requires a structured experimental design that enables to consider the concrete time course of the above outlined effects. In the present study participants therefore had to perform a Simon task in 3 blocks, each with a duration of about one hour. Between the blocks short breaks were introduced. Additionally, subjective ratings concerning mental fatigue and motivation to continue with the task were added at several time points during the experiment. In this way distinct aspects of time on task were distinguishable.

The ratings revealed that participants experienced an increase of mental fatigue and a decrease of motivation with time on task within the blocks. This is in line with other studies on long-lasting cognitive tasks^5^,^12^. However, the most intriguing result is the difference between these two measures. While the self-experience of mental fatigue showed a reset after each break almost to the initial level at the beginning of the experiment, the motivation revealed a continuous decline over all blocks. Interestingly, at the beginning of the experiment the behavioural measures showed no concordance with the time course of subjective ratings. However, there was no clear pattern concerning the relations between electrophysiological measures and the subjective ratings. During the first block no decline in performance became visible although the subjectively rated mental fatigue increased. In contrast, both the N2 as well as the P3 amplitudes showed the most prominent modulation from the first to the second sub-block. In the further time course of the experiment overall amplitude both of the N2 as well as of the P3 did not show any more great variations. We therefore were not able to fully replicate previous studies, which have shown for example a decrease of P3 amplitude caused by mental fatigue^28^,^29^. However, those studies often only included pre-post comparisons so that it is not clear whether the effects showed up at the beginning of an experiment or in the later time course. Likewise, other studies could not find a modulation of the P3 amplitude with time on task^6^. The increase of the N2 amplitude and decrease of the P3 amplitude at the beginning of the present study may therefore be caused by the adaptation on the new task^18^,^30^ or due to training effects.^31^ could find similar results for the event-related negativity (ERN), an ERP component that is related to error processing. When letting participants perform a relatively monotonous cognitive task for a long period of time, they could show the largest decrease in the ERN within the first 20 minutes of task performance. Interestingly, when providing a reward the amplitude increased again, maybe due to a novel increase in participants’ motivation. However, in the present study no clear relation between the changes in N2 or P3 amplitude and the motivation ratings could be shown.

In contrast to the ERP components, the behavioural performance decreased during the time course of the experiment, as was shown by increased response times mostly at the end of the experiment. Interestingly, at the end of the experiment a clear relation between N2 amplitude and response times could be shown. In addition, the Simon effect for error rates as well increased with time on task, which was also apparent in the N2 amplitude difference between corresponding and non-corresponding trials^6^. This leads to the suggestion that although participants already felt an increasing mental fatigue and decreasing motivation to continue with the task from the very beginning, a clear decline in behaviour was not visible before the last two blocks. This is in line with a study of 32 who showed that participants were able to perform at a good level in a cognitive task for long periods of time. However, in contrast to the study of 32 the present experiment leads to the suggestion that time on task affects participants’ ability of action control possibly because their evaluation process becomes impaired in the time course of the experiment. The ability to adequately discriminate between conflict and non-conflict trials vanishes with time on task. Therefore, participants become unable to inhibit irrelevant information (in the case of the present study the information of the stimulus location). Yet, these effects are unlikely caused by mental fatigue. It seems that the motivation to continue with the task may better explain how long participants were able to hold their performance. This is underlined by the relation between motivation, but not mental fatigue ratings and error rates in the last block and would be in line with studies, which could show that incentives were able to substantially restore the performance level in a long-lasting experiment^6^. Thus, not the exhaustion of the cognitive system, but the imbalance between the effort that is put in a given task and the expected outcome seems to determine the amount of cognitive decline^33^.

In conclusion, the present study tried to investigate the effects of time on task and mental fatigue in a long-lasting Simon task. In contrast to previous investigations we used a structured design with defined breaks and subdivided blocks that allowed interleaving subjective ratings and cognitive tests. We were able to show that at the beginning of the experiment there seems to be some unspecific modulations of training and adaptation. Time on task appears to impair participants’ ability to resolve conflict situations. In contrast to previous studies our investigation was able to reveal that the effects in a long-lasting task cannot be completely explained by mental fatigue. Instead, it seems that an interplay of adaptation at the beginning of an experiment and motivational effects in the course of the task modulates performance and psychophysiological parameters. In future studies it will therefore be important to account for the relative contribution of adaptation and motivation parameters when time on task effects are investigated.

## Methods

### Participants

14 healthy subjects (9 female) at the age of 20 to 30 years (mean = 24) participated voluntarily in this study. All of them were right-handed, non-smoking and had normal or corrected-to-normal vision. None of them had any neurological or psychiatric disorders or any sleep disorders. The participants took part in the experiment after signing an informed consent. The study was approved by the ethic committee of the Leibniz Research Centre for Working Environment and Human Factors and was conducted in accordance with the Declaration of Helsinki.

### Procedure

**Figure 6:**
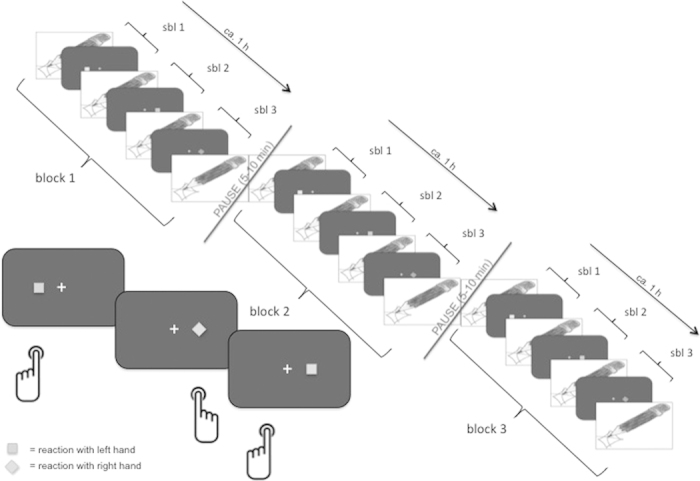
Experimental design. The experiment lasted for over 3 hours. It was divided into 3 equal blocks by short breaks of about 5 - 10 minutes. Every block was further subdivided into 3 equal sub-blocks (sbl). In each sub-block participants had to perform a Simon task which is shown in the lower left corner. Before and after the Simon task participants had to judge their current mental fatigue and motivation to continue with the task on rating scales.

The participants arrived in the laboratory at 8.30 a.m. They were instructed not to drink any coffee or black tea in the morning of the experiment. Additionally, they had to detach their watches and dispose their mobile phones. Thus they were not aware about the actual time during the whole experiment. The participants were also not informed about the duration of the study or the number of experimental blocks. At the beginning, the electrodes for the EEG were affixed and the participants were made familiar with the response buttons (custom force keys). At about one hour after arriving the participants started with the experiment. For that they were seated in a dimly lit, sound-isolated and electrically shielded cabin in front of a computer screen. The experiment consisted of 3 equal blocks (each block had a duration of about 70 minutes), which were separated by short breaks of 8 – 15 minutes (mean = 10.5, sd = 1.9) in which the participants had to leave the experimental cabin. In this time they were allowed to go to the toilet or to eat and drink. Each block was further subdivided into 3 equal sub-blocks during which participants had to perform the stimulus-response correspondence task (see below). At the beginning and at the end of each sub-block rating scales regarding the participant’s subjectively experienced fatigue and motivation to continue with the task had to be completed (see below; see Fig. 6).

### Simon task and Stimuli

The participants sat in front of a 22-inch CRT monitor (screen refresh rate of 100 Hz) with a viewing distance of 1.40 metres. The presentation of the stimuli was controlled by a VSG 2/5 graphic accelerator (Cambridge Research System, Rochester, UK). They had to look on a bright fixation cross in the middle of a dark screen (5 cd/m^2^). After an irregular interval (mean = 2800 ms) a square (■) or a diamond (♦) (10, 25 or 62 cd/m^2^, visual angle of 0.41° and 0.61° respectively) was presented for 150 ms either on the right or on the left side of the fixation cross (visual angle of 4.5° from the centre of the stimuli to the centre of fixation cross). Participants had to press the right button when the diamond appeared and the left button when the square was shown. At the same time the participants had to ignore the location where the symbol was presented (see Fig. 6). Thus, stimulus location and response side were either corresponding (stimulus and reaction on the same site) or non-corresponding (stimulus and reaction not on the same site).

### Ratings

For the participants’ subjectively experienced fatigue a slightly adapted version of the Karolinska Sleepiness Scale was used^34^. Participants had to rate their level of fatigue on a 9-step Likert scale. For the estimation of subjectively experienced motivation to continue with the task an analogical 9-step Likert scale was constructed.

### Behavioural analysis

Every press on the correct key button that occurred between 150 and 1500 ms after stimulus presentation and that exceeded 200 cN was defined as correct response. Every press on the wrong key, missed response and every press that did not comply the above mentioned criteria were defined as erroneous response. For further analysis the response time and the error rate (percentage of erroneous responses) were used.

### EEG recordings

The EEG was recorded from 60 active Ag/AgCl electrodes (ActiCap; Brain Products, Gilching, Germany) according to the 10/20 system^35^. The electrooculogram was recorded by using 2 electrodes above and below the right eye and 2 electrodes at the outer canthii of each eye. The ground electrode was fixed at AFz. As online reference an electrode at P9 was used. EEG data were processed by a BrainAmp DC amplifier (Brain Products, Gilching, Germany) using a 1000 Hz sampling rate, a notch filter at 50 Hz and a band pass filter of 0-200 Hz.

### EEG analysis

For the EEG analysis the data were re-referenced against the mastoids (TP9, TP10). The data were filtered with a high pass filter at 0.5 Hz and a low pass filter at 15 Hz. For ocular correction a regression-based algorithm according to 36 was used. Only correct responses were used for further analyses. The data were segmented in intervals of -200 ms to 800 ms after stimulus presentation. The 200 ms interval previous to stimulus presentation was set as baseline. Afterwards the data were checked for further artefacts. For ERP analysis the mean activity was measured in a time interval of 255 to 305 ms after stimulus presentation at FCz for the N2 and 475 to 525 ms at Pz for the P3 respectively. Analyses of the P3 latency were not possible because of high variability of the maximum peak between the participants, most probably due to increasing overlap by evoked alpha activity with time on task.

### Statistics

For the rating data repeated-measures-analyses of variance (ANOVA) with the factors BLOCK (bl1, bl2, bl3) and SUBBLOCK (pre, t1, t2, t3) were conducted. For the behavioural data (response times and error rates respectively) and the ERP data (N2 and P3 respectively) an ANOVA with the factors BLOCK (bl1, bl2, bl3), SUBBLOCK (sbl1, sbl2, sbl3) and CORRESPONDENCE (corr, noncorr) were conducted. For post hoc comparisons reduced ANOVAs were computed, which only included the factors of interest for further analyses of the effects.

To examine for the effects of short breaks on the subjective ratings post hoc tests between the last rating of every block and the first ratings of the next block were conducted. In addition post hoc tests between the first rating (at the beginning of the experiment) and the first ratings of the subsequent blocks were computed to investigate the potential amount of recovery after the breaks. All post hoc tests were corrected by the Bonferroni correction method.

To link the ratings with the performance and ERP data for each block correlations between the mean of the ratings and both the performance as well as normalised ERP amplitudes in the respective block were calculated. Furthermore, correlations between the normalised ERP amplitudes and the performance measures were calculated for each block separately. For significant results further correlations for each sub-block of the respective block were calculated so that a more detailed interpretation was possible.

## Additional Information

**How to cite this article**: Möckel, T. *et al.* The Effects of Time on Task in Response Selection - An ERP Study of Mental Fatigue. *Sci. Rep.* **5**, 10113; doi: 10.1038/srep10113 (2015).
